# Anti-tumor Effect of Quercetin Loaded Chitosan Nanoparticles on Induced Colon Cancer in Wistar Rats

**DOI:** 10.15171/apb.2019.048

**Published:** 2019-08-01

**Authors:** Jalil Rashedi, Amir Ghorbani Haghjo, Mehran Mesgari Abbasi, Ali Dastranj Tabrizi, Shadi Yaqoubi, Davoud Sanajou, Zahra Ashrafi Jigheh, Ali Namvaran, Ali Mohammadi, Jalal Mohammadi Khoshraj, Behzad Baradaran

**Affiliations:** ^1^Department of Biochemistry, Faculty of Medicine, Tabriz University of Medical Sciences, Tabriz, Iran.; ^2^Biotechnology Research Center, Tabriz University of Medical Sciences, Tabriz, Iran.; ^3^Drug Applied Research Center, Tabriz University of Medical Sciences, Tabriz, Iran.; ^4^Women’s Reproduction Health Research Center, Tabriz University of Medical Sciences, Tabriz, Iran.; ^5^Biotechnology Research Center and Faculty Pharmacy, Tabriz University of Medical Sciences, Tabriz, Iran.; ^6^Hematology and Oncology Research Center, Tabriz University of Medical Sciences, Tabriz, Iran.; ^7^Department of Cancer and Inflammation Research, Institute of Molecular Medicine, University of Southern Denmark, 5000 Odense C, Denmark.; ^8^Department of Analytical Chemistry, Chemistry Faculty, Tabriz University, Tabriz, Iran.; ^9^Immunology Research Center, Tabriz University of Medical Sciences, Tabriz, Iran.

**Keywords:** Anti-tumor, Chitosan, Colon, Nanoparticles, Quercetin, Rats, Wistar

## Abstract

***Purpose:*** This study was aimed to evaluate the site-specific drug delivery of 5-FU with chitosan (CS) as a carrier and quercetin (Qu) against induced colon cancer in Wistar rats.

***Methods:*** Cross-linked CS-Qu nanoparticles (NPs) were prepared by ionotropic gelation method. Physicochemical characterization of NPs was performed by Fourier-transform infrared (FTIR) spectroscopy, dynamic light scattering (DLS), in vitro drug release, and drug loading efficiency (LE). 1, 2-Dimethylhydrazine (DMH) and dextran sulfate sodium (DSS) were applied to induce adenocarcinoma tumors on inbred male Wistar rats’ colon. The treatment group of rats was administered through enema with NPs dispersion. Hematoxylin and eosin staining were performed to the histopathological examination of tumors.

***Results:*** Zeta potential and particle size for NPs were +53.5 ± 5 mV and 179 ± 28 nm, respectively. About 96% Qu LE was obtained with a maximum release of 5.63 ±1.59% and 4.62 ± 1.33% after 24 hours in PB solution with pH values of 6 and 7.4, respectively. The numbers of 8 to 21 tumors were observed in all rats administered with DMH and DSS. Significantly decreasing of microvascular density and mitosis count was detected in the treatment group in comparison with cancerous group (*P* = 0.032 for the former compared to *P* = 0.016 for the later), respectively. Furthermore, the treatment group showed a high apoptosis rate (*P* = 0.038).

***Conclusion:*** The developed Qu-loaded CS NPs were good candidates for site-specific and sustained drug release in enema treatment. Decreasing of microvascular density and mitosis count, along with increasing the apoptosis percent in the treatment group proved that the NPs could have promising results in site-specific and sustained drug delivery against colorectal cancer.

## Introduction

Colorectal cancer is the second common cancer and the fourth cause of cancer deaths in the world. In Iran, it is also the third common cancer among the men and the fourth among the women and unfortunately, with an ever-increasing rate of the disease and high mortality, it is nowadays considered as an important health issue.^1^ The most common type of colorectal cancer is adenocarcinoma^2^ which is treated with 5-fluorouracil (5-FU) as a selective chemotherapy, however it suffers from toxic effects and limited efficacy.^3,4^ In recent years, with the advent of site-specific drug delivery science for sustained and controlled release of drugs, a new pathway has been evolved to reduce the side effects and at the same time attain high efficiencies of drugs.^5,6^ Many drugs have been investigated in vitro and in vivo with various carriers. Among them, some NPs adopting chemotherapy drugs^7-9^ have been investigated whose drug delivery behavior have been considered and the satisfactory results of the effect of them on the target as well as reduced drug complications was obtained. Thanks to their low toxicity and greater biocompatibility, the utilization of natural compounds as carriers in drug delivery has attracted more attention.^10,11^ Chitosan (CS), as the only naturally occurring positive-charge polysaccharide, has remarkable properties including high bioavailability, super biodegradability, high biocompatibility, non-toxicity, etc.^12,13^ In addition to able to carry a variety of hydrophilic and hydrophobic drugs, this polymer also has the ability to open the intercellular tight junctions of epithelia.

According to the chemical structure of CS, an amine group is bonded to carbon 2 in glucose which makes the compound be a polycation in acidic environments and with capability of exhibiting the mucoadhesive feature in an encounter with the mucosa, since it contains the negatively charged sialic acids.^5^ Hence, the relatively acidic microenvironment of tumor can cause swelling of the CS nanoparticles (NPs) as well as the degradation of them by enzymes in some bacteria (especially the normal flora of the intestine). On the other hand it causes sustained release of the drug from the particle in the tumor environment.^14,15^ The release rates of various drugs have been investigated in vitro and in vivo from CS nano-microparticles (N/MPs) in different pH conditions. On the other hand quercetin (Qu), as a natural polyphenol, is the another compund that has been taken into consideration in this arena.

Qu has anti-tumor properties including antioxidant,^16-18^ anti-inflammatory,^17,19^ anti-proliferative, anti-angiogenic^20,21^ multi-drug efflux pump inhibitory,^22,23^ and pro-apoptotic,^24,25^ which elucidated in various studies. However, its in vivo anti-tumor properties especially in colorectal cancer have been less considered in animal models. Moreover, little attention has been paid on the administration of CS N/MPs as a Qu delivery system in an animal model with induced tumors and up to now, Qu has been mainly administrated without a drug delivery system. Therefore, the aim of this study is to investigate the anti-tumor effects of Qu-loaded CS NPs on induced colon cancer in male Wistar rats.

## Materials and Methods

### Materials

Chitosan (low molecular weight) was purchased from Sigma Aldrich (Germany). Quercetin Hydrate and 1, 2-Dimethylhydrazine (DMH) dihydrochloride were from TCI (Tokyo, Japan). Penta-Sodium triphosphate (Merck KGaA, Germany) was employed as cross-linker. Dextran sulfate sodium (DSS) salt (Mw 40 000; Alfa Aesar, ThermoFisher, Kandel, GmbH, Germany) was administered to induce colitis. All other chemicals were of analytical grade.

### Preparation of cross-linked CS-Qu NPs

The formulations were made by ionotropic gelation method according to the described by Xu et al^26^ with some modification. Briefly, CS (1 g) was completely dissolved in 50 ml glacial acetic acid 1%, stirred at 40°C to reach a clear solution. Qu (0.5 g) was added to the CS solution, and the dispersion was kept under stirring for 60 minutes (mixture A). 0.2 g sodium tripolyphosphate (TPP) was dissolved in 20 mL deionized water and dropped into the mixture a (1 mL/min) under vigorously mechanical stirring for 30 min at room temperature. After centrifugation in 13 000 rpm for 15 minutes and washing two-wise with deionized water, the cross-linked CS-Qu NPs were collected and dried at 40°C. The definite amount of dried nanoparticle was dispersed in deionized water at the time of administration.

### Characterization of CS-Qu NPs

#### Fourier-transform infrared (FTIR) spectroscopy analysis

The chemical structure, functional groups of Qu, CS, and the formation of blank CS NPs and Qu-loaded CS NPs were analyzed by infrared spectroscopy (Shimadzu 8400S, Kyoto, Japan) using the KBr disk method in the scanning range of 400-4000 cm^-1^, with the resolution of 2 cm^-1^.

### Zeta potential and particle size

The surface charge (zeta potential), size distribution, polydispersity index (PDI), and mean size of the Qu-loaded CS NPs were evaluated by Nano-ZS ZEN 3600 (Malvern Instruments Ltd., England) using dynamic light scattering (DLS) technique. The scattering intensity was assessed at an angle of 90° and at 25°C.

### Determination of loading efficiency (LE %)

The LE% of the NPs was calculated as the percentage ratio of the actual Qu loading of the particles to the initially Qu applied to the composite NPs. For calculation of the entrapped amount of Qu in NPs, in the final stage of the preparation of cross-linked CS-Qu NPs, after centrifugation in 13 000 rpm for 15 minutes to separate the unloaded Qu (supernatant) and Qu-loaded NPs, the unloaded Qu present was determined by an UV-Vis spectrometry (at λ_max_ of 370 nm). LE% was calculated using the following equation:

LE% = (A - B)/A × 100

Where A = Total amount of initial Qu used to prepare the NPs and B = Total amount of Qu in supernatant after centrifugation.

### In vitro drug release study

The study of in vitro release of Qu from NPs was carried out according to a previous report^27^ with minor modification. Briefly, 100 mg of dried Qu-loaded CS NPs was dispersed in 250 mL PB solutions with pH 7.4 and 6 as the simulated colon fluid (SCF) and tumor microenvironment fluid (STMF),^28,29^ respectively, at 37 ± 0.1°C and stirred in USP type I (Basket type apparatus) at 100 rpm. Then, at time intervals of 0, 1, 2, 3, 4, 5, 6, 10 and 24 h, a 1.5 ml of the solution from the release media was withdrawn with a syringe and centrifuged at 13 000 rpm for 15 minutes. The withdrawn solution was replaced with the same volume of the fresh media. Then, 1 mL of supernatant containing the released Qu was dissolved in 2 mL absolute ethanol, and its absorbance was measured using UV-Vis spectrophotometer at a wavelength of 370 nm. The amount of Qu released from the NP was calculated as a percentage of the initial amount of Qu incorporated in the NPs prior to a dissolution test.

### Experimental animals

Twenty-four inbred male Wistar rats with 90-120 g were obtained from Animal Care Center, Tabriz University of Medical Sciences and housed in cages under controlled temperature (23 ± 2 °C) and humidity, and fed a laboratory animal standard pellet diet and provided tap water *ad libitum* according to the guidelines issued by the Research Council of Tabriz University of Medical Sciences. Lighting was on a 12 h on/12 h off cycle.^30^ The animals were randomly divided into 4 groups of 6 rats in each group; they were then kept in individual wire-bottomed cages. The animals were treated as follows:

For group 1 (normal control) subcutaneous injections of normal saline were given twice a week for 20 weeks. Normal control rodents were administered through enema with 3 mL normal saline (by Nelaton catheter with 8 cm entrance from anal) daily for 3 weeks, 24 weeks after first injection of normal saline.

Group 2, the NP control group, was administered through enema with 3 ml of NPs dispersion daily for 3 weeks.

For group 3, as a cancerous control group, two carcinogen and inflammatory compounds of DMH and DSS, were applied. Animals were injected by DMH subcutaneously with 30 mg/kg body weight, twice a week for 20 weeks, after its dissolution in 1mM EDTA and adjusting the pH of final solution to 6.5 by NaOH,. After the first week of injection, 1% DSS was given in the drinking water over five days, followed by two weeks of regular water. This cycle was repeated 2 times.

Group 4, as a treatment control group, the same procedure as group 3 was adopted, and then, they were administered daily through enema with 3 mL Qu-loaded CS NPs solution, for 3 weeks, 24 weeks after first injection of carcinogen.

### Histopathological examination

After treatment period (27 weeks after first injection), the rats were anesthetized by a single injection of combined ketamine (50 mg/kg)/midazolam (1 mg/kg), and the colons were immediately removed, gently flushed with cold PBS, opened longitudinally and the tumors were fixed for 24 hours in 10% neutral buffered formalin and embedded in paraffin. Four-micrometer sections were cut for hematoxylin and eosin (H&E) staining to evaluate tumor histopathology. In addition to tumors, flat mucosa from each segment of the fixed intestine with no visible tumors was cut into 3-mm wide strips embedded in paraffin.

### Statistical analysis

The parametric variables were expressed as mean ± SD and the non-parametric ones as median (min-max). While Student’s *t* test was used for the analysis of the parametric variables. Differences were considered significant at *P* < 0.05. All statistical analyses were conducted using SPSS software (version 18).

## Results and Discussion

### Physicochemical characterization

#### FTIR

**Figure 1:**
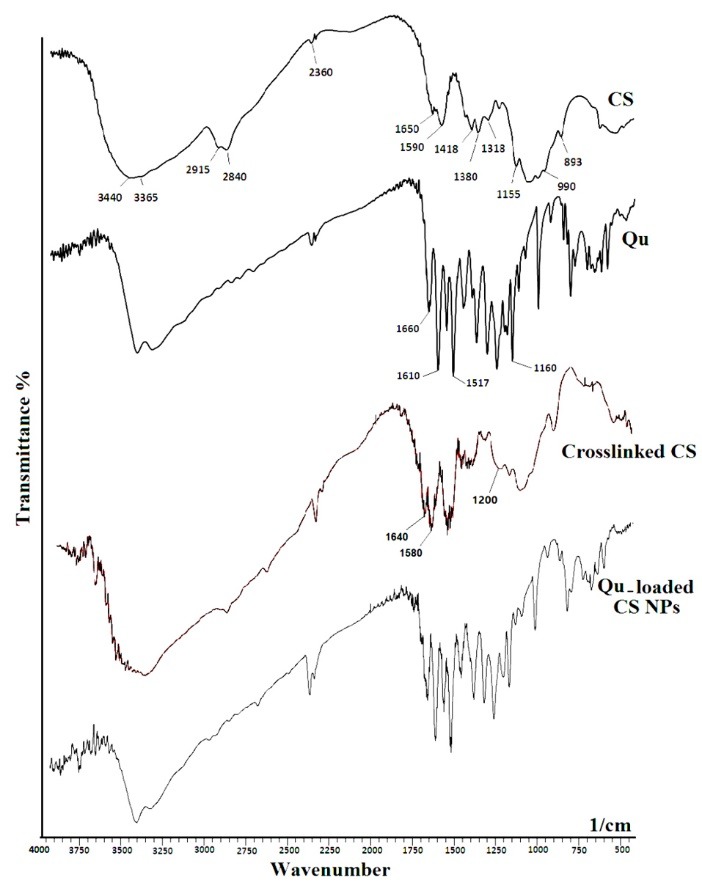
FTIR spectra of CS, Qu, cross-linked CS, and Qu-loaded CS NPs.

In order to evaluate the formation of CS NPs and to examine the probable interactions between Qu and other compounds, infrared spectroscopy was carried out. The FTIR spectra of CS, Qu, cross-linked CS, Qu-loaded CS NPs are shown in Figure 1. The CS spectrum shows typical absorption peaks at 3365, 2915, and 2840 cm^−1^, which related to –NH_2_, -CH_2_ and -CH_3_ stretching vibrations, respectively. Peaks around 1380 and 1418 cm^−1^ were attributed to the -CH_3_ and -OH vibrations, respectively. The peak around 1318 cm^-1^ was C-N stretching vibration of type I amine. The combination vibration region, 1155 to 990 cm^-1^, is related to sugar rings in the CS structure. Finally, the C–N fingerprint peak has emerged at 893 cm^−1^. One of the main peaks in CS spectrum is located in 1650 cm^-1^ related to C=O stretching of type I amide. This peak somewhat shifted to 1640 cm^-1^in the CS NPs. The enhancement in the intensity of H-bonds in CS NPs, a shift from 3440 to 3420 cm^-1^, and a broadening of peak were observed.^31^ Furthermore, the CS-NPs exhibit -N-H stretching at 1045 cm^−1^. The peaks at wavenumbers of 1590 and 1650 cm^-1^ shifted to 1580 and 1640 cm^-1^ which are related to N-H bending vibration of amine І and the amide ІІ carbonyl stretch, respectively. In CS NPs, the same band was shifted to lower wavenumbers and became broader due to the enhancing the intensity of H-bonds. A P=O peak at 1200 cm^-1^ in CS NPs can be associated to the linkage between phosphoric and ammonium ion of TPP and ammonium groups of CS.^32^

In return, the FTIR spectrum of Qu displays main unique peaks, stretching of aromatic bonds at 1160-1610 cm^−1^, and -C-O stretching at 1660 cm^−1^. A -C-C=C asymmetric stretch at 1517 cm^−1^ is present in both Qu and Qu-loaded CS NPs. The -C-O stretching at 1610 cm^−1^ also presents in the Qu-loaded CS NPs. The Qu loading in cross-linked CS can be approved by the presence of its main peaks on the spectrum of Qu-loaded CS NPs.^33^

### Zeta potential and particle size analysis

Zeta potential, the surface charge of the particles, was obtained as +53.5 ± 5 mV, which was appropriate to adhere negatively charged intestinal mucus layer^34^ as the higher the zeta potential, the greater the adhesion to the mucus. The attained proper value for zeta potential is the result of interactions of other ingredients with CS in the final structure of the particle. In some cases, the neutralizing factors such as polyanionic polymers have less effect on CS positive charge at hybridation time, so that the final charge on the particle will be more positive which is appropriate for adhesion to the mucosa.^35^ In this work, Qu, as a hydrophobic polyphenol, was successfully entrapped by cross-linked CS. A mean diameter of Qu-loaded CS particles was 179 ± 28 nm and the PDI were 0.508

### Loading efficiency (LE %)

In our study, the LE% was obtained as ~96%, so in the CS NPs the CS:Qu weight ratio can influence the rate of drug entrapment in CS N/MPs.^36^ Additionally, the type of crosslinking (chemical, physical or dual crosslinking),^37^ hybridation of CS with polyanionic/polymeric complementaries,^35^ type of hybridation (blending, coating or both of them)^31^ are the other important factors that can influence/enhance on the loading and encapsulation efficiency in drug delivery systems.

### In vitro drug release study

**Figure 2:**
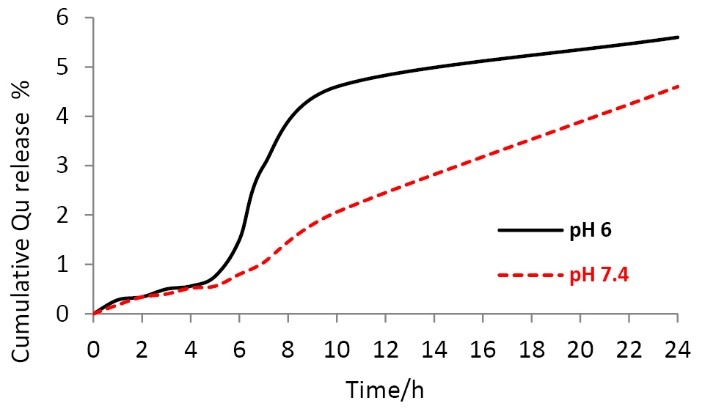
Release behavior of Qu from cross-linked CS NPs in SCF and STMF conditions.

Figure 2 and Table 1 show the release kinetics and behavior of Qu from cross-linked CS NPs in (a) simulated colon fluid (SCF, pH 7.4) compared with in (b) simulated tumor microenvironment fluid (STMF, pH 6). To determine the kinetic profile of drug release from the Qu Loaded CS NPs, release data were fitted into different kinetic models. For both pH 6 and 7.4, the release of Qu from the CS NPs was found to be best fitted into the Reciprocal power time and Peppas (power low) models with R^2^ values of 0.864 and 0.957, respectively. Also the values of ‘n’ in Peppas model (mechanism of release) were 1.142 and 1.040, which suggested that the liberation of Qu from CS NPs followed non-Fickian release kinetics. The release trend of the Qu was the same in two media up to 5 hours, but thereafter the release percentages were remarkably elevated in STMF. By decreasing of pH, the charge density of the cross-linker and therefore the cross-linking density decreases, which leads to swelling of NPs and thus release of entrapped Qu. Also, it can be related to the enhancement of CS ionization at pH ≤ 6 which relatively loses its coiled shape and becomes longer, thus it makes a high release rate.^38^

**Table 1:** The kinetics models used to fit the release data

**Kinetics model**	**pH 6**		**pH 7.4**	
	**MPE %**	**Coefficient of determination (R** ^ 2 ^ **)**	**MPE %**	**Coefficient of determination (R** ^ 2 ^ **)**
Zero order	55.04	0.805	28.65	0.984
First order	54.81	0.808	30.04	0.984
Higuchi	71.22	0.854	75.04	0.915
Peppas (Power Law)	35.12	0.865	17.63	0.957
Hixon-Crowell	54.89	0.807	29.57	0.984
Square root of mass	54.93	0.807	29.34	0.984
Three seconds root of mass	54.97	0.806	29.11	0.984
Weibull	35.08	0.865	17.84	0.957
Linear Probability	50.78	0.694	24.64	0.891
Log-Probability	37.41	0.855	22.85	0.937
Reciprocal powered time	35.04	0.864	18.04	0.956
Non-conventional order 1	55.01	0.806	28.86	0.984
Non-conventional order 2	54.77	0.809	30.24	0.984

### Histopathology

**Figure 3:**
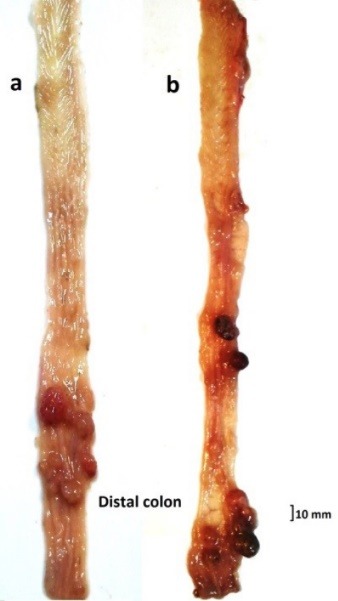
Representative macroscopic view of internal colon tissues of cancerous Wistar rats (18 cm in length); (**a**) before and (**b**) after treatment by Qu-loaded CS NPs for 3 weeks.

In all (100%) of the rats that were administered with DMH and DSS, tumors with a maximum diameter of 17 mm and minimum of 3 mm were observed (Figure 3). The number of tumors ranged from 8 to 21 in the rats often in the distal colon region. The type of all tumors were adenocarcinoma, which defined as lesions so that the neoplastic cells penetrated the muscularis mucosa to involve the submucosa or deeper layers. The depth of tumor penetration in the epithelial tissue was 91% submucosal and 9% intramuscular. Neither lymphatic vessel nor necrosis was observed in any of the treated specimens.

### Anti-angiogenic effects

**Figure 4:**
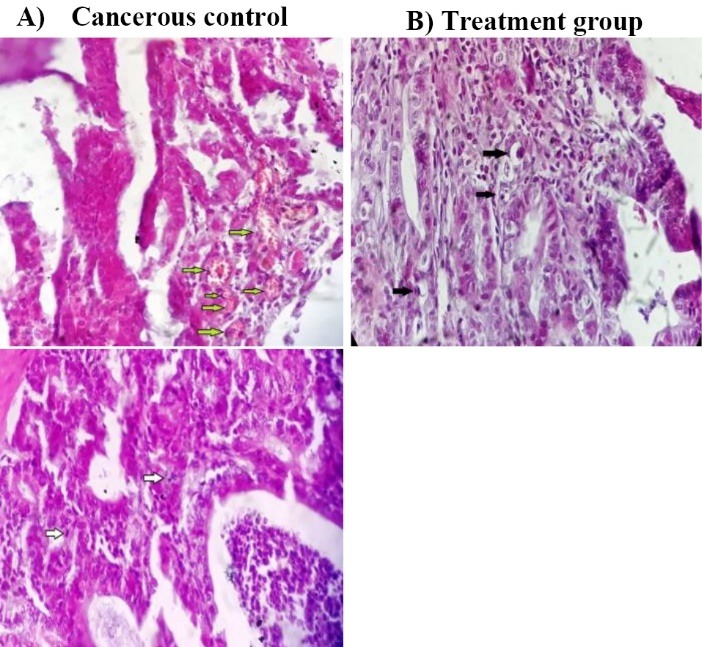
Histological sections of adenocarcinomas from Wistar rat colon. Paraffin embedded sections were stained with H&E. (A) cancerous control group, (B) treatment group; cancerous rats were administered by Qu loaded CS NPs through enema. The black arrows indicate apoptotic bodies and the white and green ones indicate mitotic cells and microvasculars, respectively (×400).

In our work, we resulted as seen in Figure 4, the results showed that Qu-loaded CS NPs could decrease significantly microvascular density in tumor tissues (*P *= 0.022). Many studies reported that Qu could inhibit several main steps of angiogenesis. The starvation, heat shock, sever exercise, and ischemia are some factors that have the ability to provoke hypoxia-induced the AMP-activated protein kinase which Qu can directly inhibit this signaling pathway.^20^ It also reduces the activity of hypoxia-inducible factor-1 and AP-1, a major transcription factor for adaptive cellular response to hypoxia and VEGF gene transcription in cancer cell lines.^21,39^ It can suppress VEGF induced phosphorylation of VEGFR_2_ and their downstream protein kinases AKT, mTOR, and ribosomal protein S6 kinase in human umbilical vein endothelial cells.^40^

### Anti-proliferative effects

Regarding the anti-proliferative effect of Qu-loaded CS NPs on tumor cells (adenocarcinoma), there was a statistically significant reduction in mitosis count of tumor cells in the treatment group in comparison with cancerous group (*P* = 0.016). Both of serine/threonine protein and tyrosine kinases, as well as phosphatases play critical roles in signal transduction pathways in order to modify the cell growth. Protein kinase could be inhibited by Qu through competing between Qu and ATP to attach the enzyme.^41^ Pathways of protein kinase C,^42^ PI3K/Akt/mTOR,^40^ PI3K/Akt/IKK-α/NF-ҠB,^43^ and mitogen-activated protein kinase ^44^ are other important signaling routes inhibited by Qu. Some studies explained that Qu could also modulate the EGFR/Akt pathway.^40^ Down regulation of Cdk1 and cyclin B in the cell cycle,^45^ and topoisomerase II are issues that attributed to Qu in proliferation.^46^ Cell cycle can be arrested by Qu at S, G2/M or G1 phases in various cancerous cell lines. This difference in phases is related to the concentration of Qu as well as the time period of the treatment.

### Pro-apoptotic effects

The previous studies showed different likely signaling pathways of Qu induced apoptosis. The capability of Qu to induce apoptosis in tumor cells, through both the intrinsic (mitochondrial) and extrinsic pathways,^24^ makes this compound as a well candidate for treatment of tumors in the delivery systems. Endoplasmic reticulum stress is the other major pathway introduced in Qu-induced apoptosis in various cancer cells.^47^ Data of this work showed a significant increase of apoptosis percent in the treatment group in comparison with cancerous one (*P* = 0.042). No remarkable increase in apoptosis in the treatment group can be obtained, probably due to the fact that the high volumes of enema solution led to rapid colon reflex and the excretion of it which results in a shorter contact time of drug. Furthermore, it causes the flushing of normal bacteria flora of colon which is needed for CS degradation and ultimately prevents the optimum release of Qu.

## Conclusion

In summary, the Qu-loaded CS NPs was prepared and employed for site-specific and sustained drug release in treatment of induced colon cancer in Wistar rats. In vitro release studies showed that little Qu is released from all NPs in SCF and STMF media; however, the total release of Qu from the NPs was higher in STMF medium due to its high swelling behavior in comparison with SCF medium. By comparing the results of angiogenesis, apoptosis, and mitosis, in cancerous group in compared to control, we resulted that the Qu-loaded CS NPs can have promising functions in site-specific and sustained drug delivery against colorectal cancers.

## Ethical Issues

Not applicable.

## Conflict of Interest

The authors declare that they have no conflicts of interest.

## Acknowledgments

This work was supported by grants from the Immunology Research Center, Tabriz University of Medical Sciences, Tabriz, Iran (thesis number 58983). The results reported in this paper are from a student thesis.
